# Co-producing a randomized controlled trial on the frequency of bathing in eczema: description of a citizen science approach

**DOI:** 10.1093/skinhd/vzaf005

**Published:** 2025-04-16

**Authors:** Arabella Baker, Natalie Bonsu, Laura Howells, Ingrid Muller, Eleanor J Mitchell, Fiona Cowdell, Firoza Davies, Mars Eddis-Finbow, Alan Montgomery, Devin Patel, Goldie Putrym, Matthew J Ridd, Miriam Santer, Amanda Roberts, Kim S Thomas, the Rapid Eczema Trials team, Kim S Thomas, Amanda Roberts, Arabella Baker, Natalie Bonsu, Tim Burton, Lucy Bradshaw, Sophia Collins, Fiona Cowdell, Firoza Davies, Mars Eddis-Finbow, Aaron Foulds, Fiona McOwan, Eleanor J Mitchell, Alan Montgomery, Ingrid Muller, Tracy Owen, Devin Patel, Tressa Goldie Putrym, Tressa Davey, Jane Ravenscroft, Shakeela Riaz, Matthew J Ridd, Miriam Santer, Hywel Williams, Kelly Zhang, Eleanor Harrison, Leila Thuman, Clare Upton, Liz Hartshorne, Nicholas Hilken, Richard Dooley, Richard Swinden, Carron Layfield, Helen Scott, Barbara Maston, Natasha Rogers, Kate Clement, Tara Dean, Angela Crooke, Philip Evans, Suzi Holland, Samantha Skelding

**Affiliations:** Centre of Evidence Based Dermatology, School of Medicine, University of Nottingham, Nottingham, UK; Faculty of Health Education and Life Sciences, Birmingham City University, Birmingham, UK; Centre of Evidence Based Dermatology, School of Medicine, University of Nottingham, Nottingham, UK; Centre of Evidence Based Dermatology, School of Medicine, University of Nottingham, Nottingham, UK; Primary Care Research Centre, School of Primary Care, University of Southampton, Southampton, UK; Nottingham Clinical Trials Unit, School of Medicine, University of Nottingham, Nottingham, UK; Faculty of Health Education and Life Sciences, Birmingham City University, Birmingham, UK; Patient and Public Contributor; Patient and Public Contributor; Nottingham Clinical Trials Unit, School of Medicine, University of Nottingham, Nottingham, UK; Patient and Public Contributor; Patient and Public Contributor; Centre for Applied Excellence in Skin & Allergy Research, University of Bristol, Bristol, UK; Primary Care Research Centre, School of Primary Care, University of Southampton, Southampton, UK; Patient and Public Contributor; Centre of Evidence Based Dermatology, School of Medicine, University of Nottingham, Nottingham, UK

## Abstract

**Background:**

Eczema is a prevalent, chronic, itchy skin condition that often persists into adulthood and significantly affects the quality of life of patients and their families. With no cure available at present, effective management is crucial. Although important patient priorities related to eczema self-management have been identified, they are rarely the focus of large, high-quality randomized controlled trials (RCTs).

**Objectives:**

To outline the methodology of using a citizen science approach to co-produce an online RCT on the frequency of bathing, to support the self-management of eczema.

**Methods:**

The co-production of the trial with patients living with eczema involved research prioritization, intervention development and trial design, all carried out through a series of online meetings and surveys.

**Results:**

Co-producing the trial took 9 months, consisting of 13 online meetings (5 to prioritize the topic, 4 to develop the intervention and 4 to design the trial), requiring 39 h of time commitment from members of the public (*n* = 12) with a total spending of £5440 on reimbursements. A prioritization survey (*n* = 120) identified the most popular research question as how often to bath/shower, receiving 49% of votes. Following an iterative refinement among the co-production group members, the trial research question was formulated. The intervention development survey (*n* = 169) established current bathing practices and interest in participating in the trial. Survey results informed the development of study materials and influenced decisions related to trial design. The finalized study materials included key information about the target behaviour (weekly bathing or daily bathing), frequently asked questions and common concerns. The trial design co-production group determined the eligibility criteria, defined the intervention and comparator, selected the outcome measures, determined the study duration and developed the recruitment strategy. The Eczema Bathing Study opened to recruitment on 29 January 2024 and over 50% of the target sample size of 390 have been recruited within the first 2 months.

**Conclusions:**

This paper provides a useful model for co-producing RCTs with members of the public. It describes the key stages of trial development (prioritization, intervention development, trial design) and contains information on the time and resources required to design trials using this approach.

## Teaser

This paper describes the co-production of an online randomized controlled trial in eczema. It provides a comprehensive description of the processes that took place to prioritize, design and conduct that trial with the involvement of patient co-production group members and the wider eczema community.

**What is already known about this topic?**

- Effective management strategies for eczema are essential.
- Key patient priorities related to self-management have been identified, but are seldom the focus of high-quality randomized controlled trials (RCTs).

**What does this study add?**

- This study describes the methodology and practicalities of co-producing an RCT with patients that addresses topics that are important to them.
- It introduces an innovative approach of combining co-production and citizen science for more relevant and rapid trials.
- It promotes inclusivity by demonstrating the value of involving patients as active stakeholders throughout the research process.

Eczema (also known as atopic dermatitis/atopic eczema) is a common, chronic, inflammatory condition that causes itchy and dry skin.^1^ It usually develops within the first 2 years of life and can persist into adulthood for many.^2,3^ Eczema can have a significant impact on quality of life for patients and their families.^4,5^ Currently, there is no cure for eczema and effective management is key. This includes avoiding irritants and triggers and using topical treatments to improve skin hydration and reduce inflammation.^6,7^

In 2013, the Eczema Priority Setting Partnership highlighted important questions for patients about skincare, washing practices, psychological interventions and the optimal use of topical treatments.^8^ Although these topics represent a high priority for patients with eczema, they are rarely the focus of large, high-quality randomized controlled trials (RCTs).^9^ Furthermore, variation in guidelines in the management of eczema and poor evidence base have also been noted.^10^ The involvement of patients as stakeholders across the research cycle is crucial to ensure that the research aligns with their priorities.^11^ In turn, this helps to improve the relevance and appropriateness of research and promote active partnership between researchers and patients.^12^ It has been shown that patient and public involvement (PPI) is particularly beneficial in RCTs.^13,14^

PPI can take many forms. Consultation involves gathering patient and public perspectives, but researchers have the ultimate decision-making power.^15^ Co-production consists of equal partnership and shared decision-making between researchers, healthcare professionals and PPI members across the research process, from defining research questions to study design and dissemination of findings.^16^ Citizen science involves the wider participation of members of the public in research activities, typically contributing to data collection and performing tasks related to the research, but they may not be directly involved in the entire research cycle.^17^

**Figure 1:**
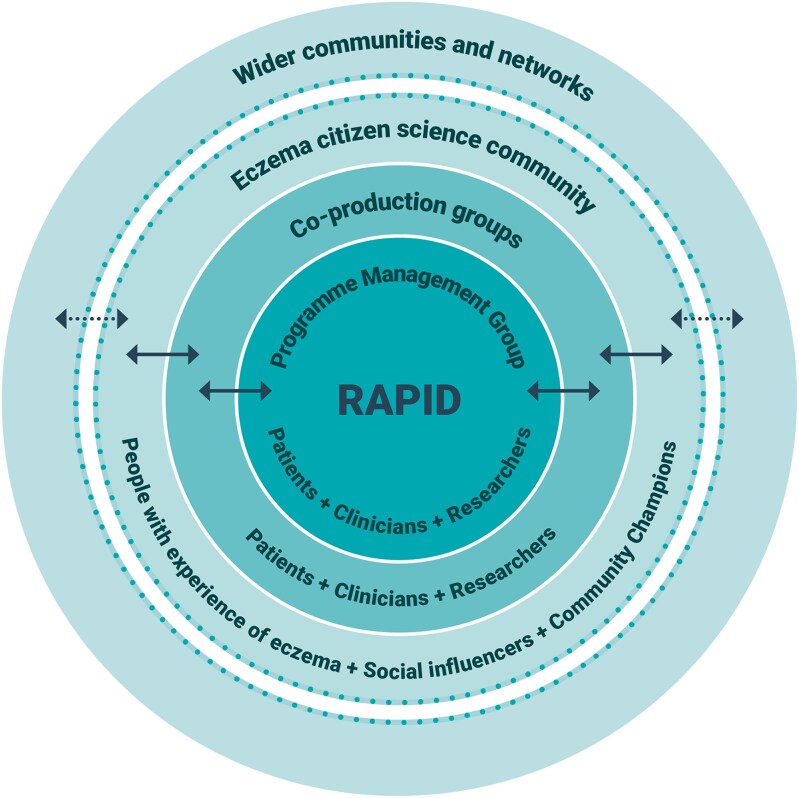
Levels of involvement in rapid eczema trials. Copyright Universtiy of Nottingham.

RCTs are the best way of comparing different interventions, but they can be time-consuming and expensive to deliver. A reformed approach to designing and conducting eczema trials is needed to generate reliable evidence in a way that is timely, relevant and cost-effective. One such innovative approach is being used in the Rapid Eczema Trials project, which includes members of the public, (i.e. ‘citizen scientists’) to prioritize, design and conduct rapid online trials that answer questions about the self-management of eczema that are important to them.^18^ This programme combines the principles of co-production and citizen science, contributing to democratizing research and promoting inclusivity that accommodates the diverse needs and perspectives of those affected by eczema.^19,20^ Individuals interested in the project can get involved in various ways as illustrated in Figure 1.

This paper describes the citizen science approach used for the co-production of the Rapid Eczema Trial's first online RCT called the Eczema Bathing Study. This trial assesses whether the frequency of bathing affects eczema severity, a topic which has not been answered by high-quality RCTs to date.^21^ Lack of evidence has resulted in conflicting advice and inconsistencies in practice that are confusing for individuals living with eczema.^22^

## Patients and methods

### Study design

#### Process evaluation of the methodology used to develop an online citizen science trial as part of the Rapid Eczema Trials project

This paper details the process of designing our first citizen science trial (Eczema Bathing Study). It summarizes key stages of trial development, the time and resources required, and key design decisions made. Participants consisted of individuals with lived experience of eczema, healthcare professionals and researchers who joined by signing up to the mailing list.

### Recruitment of co-production group members

The Eczema Citizen Science Community grew as the project was promoted through social media, snowballing within existing networks, engaging patient support groups and conducting surveys. Advertising was limited to the UK, although people were able to join the community from across the world.

Individuals with eczema and parents of children with eczema who had joined the mailing list were invited to be part of one or more of the co-production groups. The co-lead of the Rapid Eczema Trials project, who is also a patient with eczema (A.R.), hosted monthly online drop-in sessions for those interested in joining the co-production groups. The goal of these sessions was to familiarize people with the project prior to involvement and to support those with less experience in research. The co-production group consisted of volunteers, who were specifically selected to ensure diversity in relation to gender, ethnicity and age. However, initial group discussions identified the need for greater representation of male participants, non-White British individuals and younger people. To address these gaps, a targeted recruitment strategy was implemented to enhance diversity in the group.

Healthcare professionals with experience of treating eczema were identified from within the study team and via existing networks. Experts with experience of managing people with eczema throughout the care pathway were included (general practitioner, dermatologist, nurse, health psychologist).

The research team was multidisciplinary with a range of expertise in areas, including design and conduct of dermatology clinical trials; trials methodology; PPI; outcomes research; intervention development; medical statistics; online recruitment; process evaluation and community engagement.

Efforts were made to ensure that membership of the co-production groups was diverse with respect to gender, ethnicity and experience of eczema. Group members with lived experience of eczema were reimbursed for their time at rates recommended by the National Institute for Health and Care Research (NIHR) to ensure that financial constraints were not a barrier to participation. Meetings were held at a time of day agreed by the group and materials for meetings were shared via a dedicated Microsoft Teams site. In addition, a WhatsApp group was set up to enable informal conversations between group members.

### Methods of supporting co-production activities

Co-production activities were facilitated in three ways. Online co-production meetings were held via Microsoft Teams.^23^ Meetings usually lasted for 2 h, with a comfort break after 50 min. Based on the previous meeting discussions, the agenda was set by the research team along with targeted teaching and learning to ensure that co-production group members had the necessary knowledge to meaningfully engage in the decision-making process. Effective meeting facilitation was crucial for addressing potential power imbalances and ensuring productive discussions. It was achieved by using a range of facilitation techniques, including setting a well-defined agenda that was shared with all group members beforehand, leading a structured discussion; encouraging inclusive participation to ensure that everyone had the opportunity to speak and contribute; using break-out rooms and polls to gather input in various ways; summarizing information; follow-up; and documentation through the provision of meeting notes and action items after the meeting to ensure transparency, maintain accountability and reduce misunderstandings. At the first co-production meeting, ground rules for the groups were established by agreeing on a ‘ways of working roadmap’ that was subsequently used for all future meetings. Bespoke topic training was provided at the start of each meeting that was relevant to the subject under discussion.

Online surveys, mailing lists and newsletters were managed using the Mailchimp platform.^24^ The co-production surveys were open to anyone with an interest in eczema and data captured included the age, gender and ethnicity of respondents to support monitoring of diversity. Survey respondents were invited to become part of the Eczema Citizen Science Community by joining the Rapid Eczema Trials mailing list.

Monthly update newsletters were sent to subscribers to the mailing list, providing updates on project progress and opportunities to tailor their involvement according to individual interests and circumstances.

### Co-production activities

**Figure 2:**
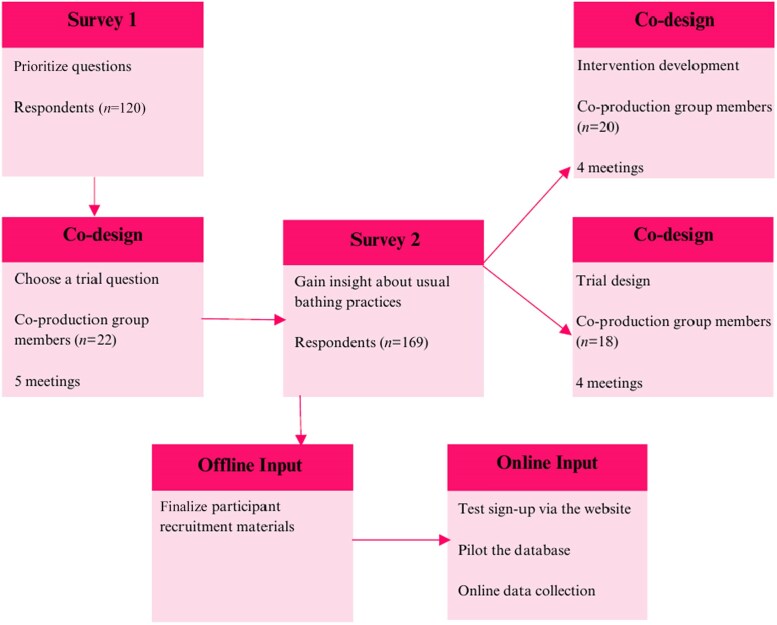
Summary of co-production.

The development of the trial included three co-production groups, each with a defined task to complete (Figure 2). As the trial was fully online, efforts were made to ensure that all trial processes were simple, inclusive and engaging. Decisions made by the co-production groups were considered against the NIHR INCLUDE Framework^7^ to ensure that inclusivity was considered at every stage of trial ­development.

#### Prioritization group

The task for the group was to prioritize the most important research question about how best to wash or bathe when having eczema.

Meetings to prioritize the topic and formulate the research question occurred every 2 weeks (Table S1; see Supporting Information). The prioritization group consisted mainly of individuals with lived experience of eczema, ensuring that the topic selection was driven by the needs of those affected by eczema rather than the preferences of researchers or healthcare professionals. Discussions were facilitated by a clinical trialist (K.S.T.) with experience in designing and running eczema RCTs.

Voting for priority topics occurred through an online survey, which was distributed to subscribers to the mailing list. The survey was also circulated to the Eczema Citizen Science Community through various channels, including social media platforms (Facebook, Instagram, TikTok and X), co-production group members’ wider networks, word of mouth and posters.

Topics included in the prioritization survey were based on the topics of the James Lind Alliance Priority Setting Partnership.^7^ Survey respondents were asked to select their top three research questions to be answered and had the option to suggest additional topics in free text. Results were summarized as a frequency vote for each of the included topics.

Prioritization by the co-production group was based on: (i) voting results from the online survey; (ii) feasibility of conducting an online trial on the topic; and (iii) evidence that there was a research gap on the topic.

#### Intervention development group

The task for the group was to develop inclusive, theory-informed study materials that would encourage adherence to the two study groups (weekly bathing and daily bathing).

Meetings to develop the intervention and related study materials took place every 2–4 weeks (Table S2; see Supporting Information) and were held in parallel with the trial design co-production meetings. This ensured that aspects arising from discussions about trial design were considered and reflected in the study materials. Discussions were facilitated by a health psychologist (I.M.) with experience of developing self-management behavioural interventions for eczema. Intervention development followed the principles of the person-based approach to gauge people's perspectives on the behavioural components of the intervention.^25^

To ascertain current bathing practices of people with eczema, the intervention development survey was developed. The survey questions helped to establish barriers and facilitators to changing bathing practices and willingness to take part in a future RCT. The survey was distributed in a similar manner to the prioritization survey detailed above. Members of the intervention development co-production group developed the study materials to ensure the messages were meaningful and appropriate, using simple language throughout.

#### Trial design group

The task for the group was to design an online RCT, comparing weekly bathing to daily bathing for the management of eczema.

Meetings to design the trial took place every 2–4 weeks and ran in parallel with the intervention development co-production meetings. Details of topics included in the trial design meetings are summarized in Table S3 (see Supporting Information). Discussions were facilitated by clinical trialists (E.J.M. and K.S.T.) with experience of designing and conducting RCTs.

The co-production group was required to make decisions on the following aspects of the trial: (i) eligibility criteria; (ii) intervention and comparator (building on work by the prioritization and intervention development co-production groups); (iii) outcomes; (iv) trial duration; and (v) recruitment strategy (ensuring wide reach and inclusivity).

A summary of the decisions made by the three co-­production groups and copies of the study materials were supplied to the Nottingham Clinical Trials Unit (NCTU) who were managing trial delivery. NCTU staff used this information to generate the study protocol, recruitment materials, obtain ethical approval and develop the study database, prior to opening the trial for recruitment.

A process evaluation to gauge the success of this co-­production approach to trial development is ongoing and will be reported separately.

### Patient and public involvement

In this citizen science project, PPI has been embedded throughout. Examples of changes made to the trial following PPI feedback include ensuring trial participants were not financially disadvantaged (e.g. if asked to have a bath or shower more often than normal) and that payment methods were handled with sensitivity and minimal paperwork; addressing challenges related to online consent processes for people < 16 years old; helping to understand the advantages and disadvantages of translating materials into other languages vs. using simple language accessible to people who do not speak English as their first language; highlighting cultural differences in bathing practices and ensuring that study materials were inclusive; and testing the database prior to trial launch. Through the knowledge mobilization group, PPI will facilitate dissemination of study results to those who need it.

## Results

### Delivery of the co-production groups

In total, 12 patients and carers joined the prioritization co-production group. Most of them chose to remain involved throughout the process, resulting in an overlap of membership across the three groups.

In total 13 co-production meetings were held between 7 November 2022 and 27 July 2023, taking 9 months to develop the trial. All meetings occurred on Thursday evenings from 5 to 7 pm, which was the most convenient time for group members with lived experience of eczema. This schedule represented a time commitment of 26 h for the online co-production meetings and approximately 13 h of meeting preparation. A total of £5440 was spent on reimbursing PPI group members for their time. Additionally, £217.97 was spent to promote the project via paid advertisements on social media platforms.

### Prioritization of research topic

The research prioritization survey was open from 9 September 2022 to 30 January 2023. In total, 120 respondents voted for their top research questions. Most respondents (65%) were aged 26–59 years, White British (*n* = 69/120; 57.5%) and had eczema themselves (Table S4; see Supporting Information). Results of the survey are shown in Table 1. The most popular research questions were: (i) ‘How often should people with eczema have a bath or shower?’ (49% votes); and (ii) ‘Are nonbiological washing powders better than biological washing powders for washing the clothes of people with eczema?’ (33% votes).

**Table 1:** Results of the research prioritization survey [responders (*n* = 120) asked to choose their top three topics]

Research questions (each respondent could choose up to three from this list)	Responses, *n* (%)
How often should people with eczema have a bath or shower?	59 (49.2)
Are nonbiological washing powders better than biological washing powders for washing the clothes of people with eczema?	39 (32.5)
Does bathing in cooler water improve eczema compared to hotter water temperatures?	28 (23.3)
Are baths better than showers for eczema?	26 (21.7)
Do salt baths help eczema?	24 (20.0)
Does avoiding soap whilst bathing improve eczema?	24 (20.0)
What is the best way to protect the skin when swimming?	20 (16.7)
Should moisturizers (emollients) be used after bathing?	17 (14.2)
How long should you bathe or shower for?	16 (13.3)
Does avoiding shampoo in the bath/shower help eczema?	15 (12.5)
Does exfoliating while bathing help eczema or make it worse?	15 (12.5)
Do bleach baths help infected eczema?	15 (12.5)
Does removal of chlorine or water hardness improve eczema?	15 (12.5)
Do antibacterial bath products help infected eczema?	14 (11.7)
Should moisturizers (emollients) be used before and after bathing?	12 (10.0)
How often should bedding be changed to avoid build-up of bacteria?	12 (10.0)
How long after bathing should you apply flare control creams?	9 (7.5)
What is best for washing hands – normal soap antibacterial soap or antibacterial gel?	9 (7.5)
Are nonsoap washing options (e.g. ecoballs) better than soap washing powders?	7 (5.8)
What is the best way to shave with eczema?	4 (3.3)
Do chickweed baths help eczema?	3 (2.5)

The research prioritization co-production group consisted of 12 people with experience of eczema, 6 researchers and 4 healthcare professionals. A total of five meetings were held across 2.5 months (7 November 2022 to 19 January 2023). Based on the results of the prioritization survey and through an iterative refinement among the co-production group members, the trial research question was formulated: ‘In people (adults and children) with mild to moderate eczema, is less frequent (1 or 2 times per week) bathing better than frequent (6 or 7 times per week) bathing?’

### Intervention development

The intervention development co-production group included 12 people with experience of eczema, 5 researchers and 3 healthcare professionals. In total, four meetings took place across 5 months (16 February to 8 June 2023).

The intervention development survey was open between 9 March 2023 and 6 May 2023 and received 169 responses. Most respondents were female (*n* = 111/169; 65.7%), White British (*n* = 120/169; 71.0%) and had self-reported eczema (*n* = 107/169; 63.3%) (Table S4). Table 2 shows the survey responses and a summary of how these responses influenced intervention development and trial design decisions. Study materials were developed iteratively by the group based on results of the survey and discussions within the group. The finalized materials included key information about the target behaviour (weekly bathing or daily bathing), frequently asked questions and common concerns. A behavioural analysis was carried out to map the key barriers to weekly or daily bathing to intervention content and behaviour change techniques were used to address these.

**Table 2:** Results of the intervention development survey and impact on design

Questions and response options	Responses (*n* = 169)	Impact on intervention development	Impact on trial design
Do you most often have a bath or a shower? (*n* = 168)		Study materials to mention both bath and shower	Research question addresses any kind of bathing that involves whole-body washing
Shower	90 (53.5)	Allow flexibility for participants to use both bath and/or shower	
Bath	48 (28.5)	Include ‘Other’ option in responses	
Regularly use both showers and baths	29 (17.2)		
Other	1 (0.5)		
How many days per week do you usually have a bath or shower? (*n* = 169)		Added FAQs to help participants change their bathing practices during the trial	Daily bathing chosen as ‘normal practice’ group
Every day (at least once per day)	72 (42.6)		Weekly bathing chosen as ‘intervention group’
6 days per week	9 (5.3)		Daily bathing defined as bathing ≥ 6 times weekly
5 days per week	14 (8.2)		Weekly bathing defined as bathing 1 or 2 times weekly
4 days per week	23 (13.6)		
3 days per week	21 (12.4)		
2 days per week	17 (10.0)		
Once a week	8 (4.7)		
Other	5 (2.9)		
Do any of the following influence how often you have a bath or shower? (Select all that apply) (*n* = 297)^a^		Financial support available to help cover costs related to taking part in the study	Trial duration of 4 weeks to limit potential financial burden and commitment required to take part
Cost of wash products	4 (1.3)		Offer payment to both groups to ensure equal treatment of groups as much as possible
Cost of bills	5 (1.6)		
Bathing/showering helps my eczema	51 (17.1)		
Bathing/showering worsens my eczema	54 (18.1)		
Enjoyment/relaxation	51 (17.1)		
Sweat a lot	17 (5.7)		
Do not sweat a lot	13 (4.3)		
It is a routine I have got used to doing	77 (25.9)		
Other	25 (8.4)		
How long do you usually spend in the bath or shower? (*n* = 169)		FAQs added to intervention materials about maintaining the usual duration of shower or bath	Nothing else to be changed apart from frequency of bathing
Less than 5 min	23 (13.6)		
5–10 min	65 (38.5)		
11–20 min	52 (30.8)		
21–30 min	13 (7.7)		
Over 30 min	12 (7.1)		
Other	4 (2.4)		
What products do you usually use in the bath or shower? (Select all that apply) (*n* = 300)^a^		FAQs added to intervention materials about using bathing products as usual	Nothing else to be changed apart from frequency of bathing
None, only water	18 (6.0)		
Shower gel (regular)	16 (5.3)		
Shower gel (for sensitive skin)	58 (19.3)		
Shower gel (for babies)	7 (2.3)		
Shampoo/conditioner	74 (24.6)		
Bubble bath	10 (3.3)		
Moisturizing creams (emollients)	80 (26.6)		
Other	37 (12.3)		
What products do you apply to your skin after bathing/showering? (Select all that apply) (*n* = 255)^a^		FAQs added to intervention materials about using topical treatments as usual	Nothing else to be changed apart from frequency of bathing
None	10 (3.9)		
Moisturizing creams (emollients)	144 (56.1)		
Other eczema treatments (e.g. flare control creams)	91 (35.9)		
Other	10 (3.9)		
Would you be willing to change how often you have a bath or shower whilst taking part in a study? (*n* = 166)		Study materials to emphasize the importance of following the allocated bathing frequency	Screening question for eligibility
Yes	82 (49.3)		
No	37 (22.2)		
Not sure	47 (28.3)		
Would you be willing to have a bath or shower most days (6 or 7 times a week) whilst taking part in a study? (*n* = 164)		Informed preparation of intervention materials	Supported decision to select daily bathing ‘normal practice’ group
Yes	120 (73.1)		
No	27 (16.4)		
Not sure	17 (10.3)		
Would you be willing to have a bath or shower only once or twice a week whilst taking part in a study? (*n* = 164)		Can wash using flannel/sponge in the sink in between showers or baths	Allow to wash without baths or showers
Yes	74 (45.1)	Can wash hair in between showers or baths	
No	63 (38.4)		
Not sure	27 (16.4)		
How long would you be willing to stay in a study where you would be asked to change how often you have a bath or shower? (*n* = 155)		Helped to establish people’s attitudes about changing bathing habits	4 weeks, study duration with weekly data collection
Would not take part	39 (25.1)		
Less than 2 weeks	10 (6.4)		
2–3 weeks	17 (10.9)		
4–5 weeks	13 (8.3)		
6 weeks	24 (15.4)		
As long as needed to answer the research question	52 (33.5)		

Data are presented as n (%) unless otherwise stated. FAQ, frequently asked questions. ^a^Respondents could choose more than one answer.

The written study materials agreed by the co-production group were used to create infographics, written information and short videos with subtitles. This approach aimed to offer information in a variety of engaging and accessible formats. The developed materials can be viewed in Supplemental File S1 (see Supporting Information).

### Trial design

In the trial design co-production group 10 patients and 8 researchers were involved. A total of four meetings were held over 4 months (27 April to 27 July 2023). These meetings were evenly spaced, although there was some overlap with the intervention development meetings. However, both meetings were well attended by co-production group members. The key decisions made about the trial by this group are shown in Table 3.

**Table 3:** Decisions made by the trial design co-production group

Trial design aspect	Decision	Rationale
Inclusion criteria	Aged ≥1 year with self-report of eczema	Ensures broad inclusivity and generalizibility
	Usual residence in the UK	Indemnity cover relates to UK only
	Able and willing to give informed consent (or parent/legal guardian able and willing to give informed consent for children aged under 16 years)	Following ethical research practices
Exclusion criteria	None or very mild eczema symptoms	Allows measurement of intervention effectiveness avoiding floor effects
	Eczema only present on hands (likely to be hand eczema or contact dermatitis); limited to locations where skin exposed to nickel, e.g. jewellery (likely to be contact dermatitis); or eczema only around varicose veins (likely to be varicose eczema)	Focus on atopic eczema, not contact dermatitis or varicose eczema
	Started a new eczema treatment (including antibiotics for eczema) other than emollients in the last 4 weeks	Confounding factor
	Taking part in another eczema intervention trial	Confounding factor
	Unable or unwilling to change bathing practices for 4 weeks	Adherence to group allocation required
	Planning to swim more than twice a week in the next 4 weeks (including surfing, scuba diving, etc.)	Frequent swimming introduces additional water exposure that could affect eczema severity
	Member of household already participating in this trial	Potential for shared behaviours or influences that might affect trial outcomes
Intervention group	Bathing no more than 1 or 2 times per week	Based on survey results
Control group	Bathing 6 or more times per week	Was deemed more feasible by the co-production group
Primary outcome	Eczema severity, assessed by the POEM instrument	Commonly used, well-validated, patient-reported eczema severity measure that is sensitive to change
Secondary outcomes	Itch intensity in the last 24 h	Part of the eczema core outcome set
	Quality of life	Part of the core outcome set
	Eczema control	Part of the core outcome set
	Use of usual topical treatments for eczema	Helps to establish change in treatment use
	Proportion of participants who achieve an improvement in POEM at week 4 of ≥3.0 points compared with baseline	Ensures change in POEM scores is clinically meaningful
	Global assessment of eczema	Global eczema severity measure
	Adverse events—need to see a healthcare professional due to worsening of the eczema	Safety monitoring
Trial duration	4 weeks	Changing bathing habits for a shorter time period is more acceptable and helps to reduce attrition
Recruitment strategy	Rapid Eczema Trials mailing list	Subscribers to the project
	Social media (posts and paid adverts)	Broad and targeted reach, tailored to needs
	Database search and mailout by GP surgeries	Reach of diverse communities
	Eczema charities	Significant number of active followers
	Outreach and engagement events	Face to face contact with potential participants
	NIHR Be Part of Research website	List of patients interested in eczema research
	Posters and flyers	Promoting the study through offline methods
	Snowballing through the Eczema Citizen Science Community	Word of mouth can reach those not present on social media platforms or events

POEM, Patient Oriented Eczema Measure; GP, general practitioner; NIHR, National Institute for Health and Care Research.

## Discussion

This paper describes the methodology of using a citizen science approach for co-producing an online eczema RCT to evaluate the impact of weekly bathing compared to daily bathing on eczema severity. The co-production process took 9 months to complete and consisted of 13 meetings. An approximately 39-h time commitment was required from each member of the public, with a total cost of £5440 to compensate for their time. The views of the Eczema Citizen Science Community were captured through two online surveys and the results were used to inform decision-making. Study materials were developed using a theory-informed, person-based approach and were co-produced to ensure usability and accessibility among diverse communities.

People with lived experience of eczema played an active role as equal partners and contributed to decision-making throughout the project. The co-production process was flexible and evolved to suit the needs of the groups. The regular bi-weekly meetings for the trial development stages and the informal WhatsApp group helped to foster a collegiate and supportive environment for the project.

The benefits of co-producing the Eczema Bathing Study and its impact on trial success criteria will be explored through an ongoing process evaluation focusing on speed of recruitment, diversity of trial participants, retention and adherence to intervention. Recruitment for the trial began on 29 January 2024 and initial signs are encouraging. Within the first 2 months, > 50% of the target sample size of 390 participants was recruited. All progression criteria for the internal pilot phase were successfully met, suggesting that this co-produced trial with citizen scientists was able to avoid many of the difficulties in trial recruitment and retention commonly experienced in RCTs.^26–29^ On completion of the trial, members of the co-production groups will also contribute to the interpretation of the results, dissemination and knowledge mobilization activities.

This project provided opportunities for people with eczema to actively contribute to prioritizing, designing and conducting an online RCT. Those involved gained insights into research, learnt new skills, were challenged to think differently and worked to build a community of like-minded individuals who share the common belief that the lives of patients can be improved through better quality, patient-centred research. The co-production group members represented broad geographical, socioeconomic and cultural diversity.

This project relied on digital communication methods, which may have inadvertently excluded some individuals. However, it facilitated wide geographical coverage and flexibility as people were able to fit meetings around existing commitments and did not need to travel. Reasonable adjustments were made for those who found it challenging to engage in online meetings (e.g. by posting printed materials of long documents and creating inclusive and supportive environments for neurodiverse individuals).

Most co-production meetings (*n* = 12/13; 92%), were held in the evenings, outside standard office hours, to accommodate the availability of public members. This required researchers and healthcare professionals to frequently work beyond their usual working hours, potentially compromising work–life balance.

Active involvement of members of the public in all aspects of the research cycle is strongly recommended by research funding bodies and policy makers.^10,30^ Thus, the co-production of patient-centred research is becoming increasingly common.^31,32^

The Rapid Eczema Trials project is unique in its emphasis on co-producing a series of RCTs aimed at addressing issues related to eczema self-management. Learning from the Eczema Bathing Study will inform subsequent RCTs within this programme of research. As part of this approach, we are continuously building an Eczema Citizen Science Community which includes people interested in contributing to the trials and sharing the results. This is helping to facilitate wider engagement and input from different social groups and communities, enabling the rapid and broad dissemination of trial findings.

A similar approach to prioritizing, designing and sharing results of an online RCT was used in The People's Trial (https://thepeoplestrial.ie/), but the aim of this study was to enhance the public's understanding of RCTs rather than to answer clinically important questions in a specific disease area. The People's Trial included 3000 participants from 72 countries who engaged in different aspects of the trial.^33^ Similarly, in studies of long COVID various forms of PPI involvement occurred including a citizen science approach to identify research priorities,^34^ and, more recently, a co-production methodology was used for a feasibility trial of non-pharmacological interventions.^35^ All studies reported ongoing engagement and input from the public involved, highlighting the plausibility and value of this approach.

The Eczema Bathing Study is the first of several eczema trials that will be co-produced through the Rapid Eczema Trials project. This innovative approach to developing multiple co-designed trials in eczema means that learning, resources and procedures developed for our first trial can be reused for subsequent trials. Consequently, certain trial processes (such as data collection and ethical approval procedures) can be optimized allowing for the delivery of increasingly efficient eczema trials throughout the project. Additionally, trial resources will be openly shared with other researchers and members of the public wishing to run their own eczema trials.

This paper provides a useful case study for others interested in co-producing RCTs with members of the public. It presents information on the time and resources required to design trials using this approach. Our surveys offer insights into topics important to individuals living with eczema around washing and current bathing practices in the UK, which can inform future trials in this area.

## Supplementary Material

vzaf005_Supplementary_Data

## Data Availability

Deidentified participant level data will be available on reasonable request to the corresponding author.

## Appendix 1Contributors to the eczema bathing study

Co-production group members: Kim S. Thomas (lead), Amanda Roberts (lead, patient), Arabella Baker (researcher), Natalie Bonsu (researcher), Tim Burton (patient), Lucy Bradshaw (researcher), Sophia Collins (researcher), Fiona Cowdell (healthcare professional), Firoza Davies (patient), Mars Eddis-Finbow (patient), Aaron Foulds (patient), Fiona McOwan (patient), Eleanor J. Mitchell (researcher), Alan Montgomery (researcher), Ingrid Muller (researcher), Tracy Owen (patient), Devin Patel (patient), Tressa Goldie Putrym (carer), Tressa Davey (patient), Jane Ravenscroft (healthcare professional), Shakeela Riaz (carer), Matthew J. Ridd (carer, researcher and healthcare professional), Miriam Santer (healthcare professional), Hywel Williams (healthcare professional), Kelly Zhang (carer)

Project management and delivery: Eleanor Harrison, Leila Thuman, Clare Upton, Liz Hartshorne

Database development: Nicholas Hilken, Richard Dooley, Richard Swinden

Programme manager and PPI lead: Carron Layfield

Administrative and technical support: Helen Scott, Barbara Maston, Natasha Rogers, Kate Clement

Independent steering group members: Tara Dean, Angela Crooke, Philip Evans, Suzi Holland, Samantha Skelding

## References

[1] Bieber T . Atopic dermatitis. Ann Dermatol 2010; 22:125–37. 20548901 10.5021/ad.2010.22.2.125 PMC2883413

[2] Wollenberg A, Oranje A, Deleuran M et al ETFAD/EADV Eczema Task Force 2015 position paper on diagnosis and treatment of atopic dermatitis in adult and paediatric patients. J Eur Acad Dermatol Venereol 2016; 30:729–47. 27004560 10.1111/jdv.13599

[3] Margolis JS, Abuabara K, Bilker W et al Persistence of mild to moderate atopic dermatitis. JAMA Dermatol 2014; 150:593–600. 24696036 10.1001/jamadermatol.2013.10271 PMC4352328

[4] Maksimović N, Janković S, Marinković J et al Health-related quality of life in patients with atopic dermatitis. J Dermatol 2012; 39:42–7. 22044078 10.1111/j.1346-8138.2011.01295.x

[5] Silverberg JI, Gelfand JM, Margolis DJ et al Patient burden and quality of life in atopic dermatitis in US adults: a population-based cross-sectional study. Ann Allergy Asthma Immunol 2018; 121:340–7. 30025911 10.1016/j.anai.2018.07.006

[6] National Institute for Health and Care Excellence . Atopic eczema in under 12s: diagnosis and management 2007. Available at: https://www.nice.org.uk/guidance/CG57/chapter/1-Guidance#treatment (last accessed 17 February 2025). 34101396

[7] Batchelor JM, Ridd MJ, Clarke T et al The Eczema Priority Setting Partnership: a collaboration between patients, carers, clinicians and researchers to identify and prioritize important research questions for the treatment of eczema. Br J Dermatol 2013; 168:577–82. 22963149 10.1111/bjd.12040

[8] Nankervis H, Thomas KS, Delamere FM et al Scoping Systematic Review of Treatments for Eczema. Southampton, UK: NIHR Journals Library, 2016. 27280278

[9] Van Halewijn KF, Lahnstein T, Bohnen AM et al Recommendations for emollients, bathing and topical corticosteroids for the treatment of atopic dermatitis: a systematic review of guidelines. Eur J Dermatol 2022; 32:113–23. 35188464 10.1684/ejd.2022.4197

[10] Staniszewska S, Adebajo A, Barber R et al Developing the evidence base of patient and public involvement in health and social care research: the case for measuring impact. Int J Consum Stud 2011; 35:628–32.

[11] National Institute for Health and Care Research . Briefing notes for researchers—public involvement in NHS, health and social care research 2021. Available at: https://www.nihr.ac.uk/documents/briefing-notes-for-researchers-public-involvement-in-nhs-health-and-social-care-research/27371 (last accessed 17 February 2025).

[12] Boote J, Baird W, Sutton A. Public involvement in the design and conduct of clinical trials: a review. Int J Interdiscip Social Sci 2011; 5:91–111.

[13] Crocker JC, Ricci-Cabe I, Parker A et al Impact of patient and public involvement on enrolment and retention in clinical trials: systematic review and meta-analysis. BMJ 2018; 363:k4738. 30487232 10.1136/bmj.k4738 PMC6259046

[14] Gibbins KJ, Lo JO. What matters to whom: patient and public involvement in research. Clin Obstet Gynecol 2022; 65:268–76. 35476620 10.1097/GRF.0000000000000694 PMC9060323

[15] Hickey G, Brearley S, Coldham T et al Guidance on co-producing a research project. Southampton: INVOLVE, 2018. Available at: https://www.learningforinvolvement.org.uk/wp-content/uploads/2021/04/NIHR-Guidance-on-co-producing-a-research-project-April-2021.pdf. (last accessed 11 March 2025).

[16] Woolley JP, McGowan ML, Teare HJA et al Citizen science or scientific citizenship? Disentangling the uses of public engagement rhetoric in national research initiatives. BMC Med Ethics 2016; 17:33. 27260081 10.1186/s12910-016-0117-1 PMC4893207

[17] Howells L, Muller I, Baker A et al Can an online approach to citizen science revolutionise clinical trials? Eur Health Psychol 2024; 23:1103–9.

[18] King AC, Winter SJ, Chrisinger BW et al Maximizing the promise of citizen science to advance health and prevent disease. Prev Med 2019; 119:44–7. 30593793 10.1016/j.ypmed.2018.12.016 PMC6687391

[19] Butler CC . Democratising the design and delivery of large-scale randomised, controlled clinical trials in primary care: a personal view. Eur J Gen Pract 2024; 30:2293702. 38180050 10.1080/13814788.2023.2293702 PMC10773679

[20] Hua T, Yousaf M, Gwillim EC et al Bathing practices in atopic dermatitis: a systematic review and meta-analysis. J Am Acad Dermatol 2020; 83(Suppl):AB199.

[21] Teasdale E, Muller I, Sivyer K et al Views and experiences of managing eczema: systematic review and thematic synthesis of qualitative studies. Br J Dermatol 2021; 184:627–37. 32531800 10.1111/bjd.19299

[22] Microsoft Teams . Microsoft Teams for desktop 2024. Available at: https://www.microsoft.com/en-gb/microsoft-teams/download-app#download-for-desktop (last accessed 17 February 2025).

[23] Mailchimp . Turn emails into revenue 2024. Available at: https://tinyurl.com/f7zbsyvj (last accessed 17 February 2025).

[24] National Institute of Health Research . The INCLUDE Ethnicity Framework 2020. Available at: https://www.trialforge.org/trial-­diversity/include/ (last accessed 17 February 2025).

[25] Yardley L, Morrison L, Bradbury K, Muller I. The person-based approach to intervention development: application to digital health-related behavior change interventions. J Med Internet Res 2015; 17:e30. 25639757 10.2196/jmir.4055 PMC4327440

[26] Gul RB, Ali PA. Clinical trials: the challenge of recruitment and retention of participants. J Clin Nurs 2010; 19:227–33. 20500260 10.1111/j.1365-2702.2009.03041.x

[27] Fletcher B, Gheorghe A, Moore D et al Improving the recruitment activity of clinicians in randomised controlled trials: a systematic review. BMJ Open 2012; 2:e000496. 10.1136/bmjopen-2011-000496 PMC3253423 22228729

[28] Kasenda B, von Elm E, You J et al Prevalence, characteristics, and publication of discontinued randomized trials. JAMA 2014; 311:1045–52. 24618966 10.1001/jama.2014.1361

[29] Walters SJ, dos Anjos Henriques-Cadby IB, Bortolami O et al Recruitment and retention of participants in randomised controlled trials: a review of trials funded and published by the United Kingdom Health Technology Assessment Programme. BMJ Open 2017; 7:e015276. 10.1136/bmjopen-2016-015276 PMC5372123 28320800

[30] National Institute for Health and Care Research . How to involve the public in knowledge mobilisation. Available at: https://evidence.nihr.ac.uk/collection/how-to-involve-the-public-in-knowledge-­mobilisation/ (last accessed 17 February 2025).

[31] Slattery P, Saeri AK, Bragge P. Research co-design in health: a rapid overview of reviews. Health Res Policy Syst 2020; 18:17. 32046728 10.1186/s12961-020-0528-9 PMC7014755

[32] McCarron TL, Clement F, Rasiah J et al Patients as partners in health research: a scoping review. Health Expect 2021; 24:1378–90. 34153165 10.1111/hex.13272 PMC8369093

[33] Finucane E, O’Brien A, Treweek S et al The People’s Trial: supporting the public’s understanding of randomised trials. Trials 2022; 23:205. 35264220 10.1186/s13063-021-05984-1 PMC8905031

[34] Ziegler S, Raineri A, Nittas V et al Long COVID citizen scientists: developing a needs-based research agenda by persons affected by long COVID. Patient 2022; 15:565–76. 35478078 10.1007/s40271-022-00579-7 PMC9046008

[35] Turner GM, McMullan C, Aiyegbusi OL et al Co-production of a feasibility trial of pacing interventions for long COVID. Res Involv Engagem 2023; 9:18. 36997975 10.1186/s40900-023-00429-2 PMC10061378

