# Supplementary material for: Co-producing a randomized controlled trial on the frequency of bathing in eczema: description of a citizen science approach
Source: Skin Health Dis. 2025 Apr 16;5(2):130–9. doi: 10.1093/skinhd/vzaf005 (PMC12068486; doi:10.1093/skinhd/vzaf005)
Supplement: vzaf005_Supplementary_Data [file vzaf005_supplementary_data.zip › Supplemental File.pdf]

Nottingham University Hospitals NHS Trust

Rapid Eczema Trials — Creating research together

University of Nottingham UK | CHINA | MALAYSIA

Eczema Bathing Study

Thank you for taking part.

You are ready to go!

You are in the Daily Bathing group…

bath or shower 6 or more times per week

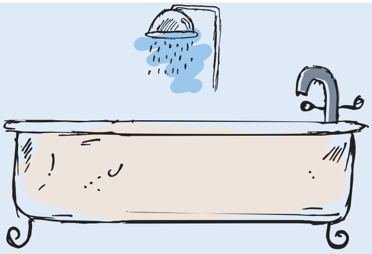

You are in the group that will have a bath or shower 6 or more times per week for the next 4 weeks. A bath or shower includes any way of washing that soaks your body in water.

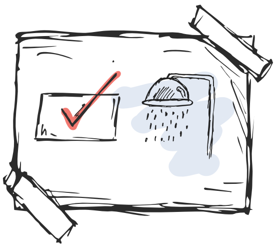

It is important to stick to having a bath or shower 6 or more times per week.

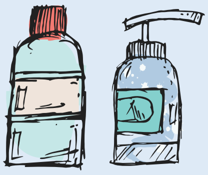

You don't need to change anything else about the way you wash or manage your eczema.

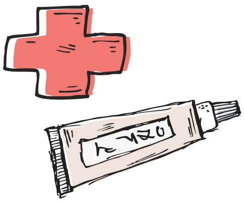

This does not affect your usual eczema care. You can consult your GP, pharmacist or other care provider as usual. If possible, do not start any new eczema treatments for the next 4 weeks.

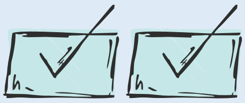

You will fill in questionnaires every week. These are important to help understand how you are getting on.

Frequently Asked Questions

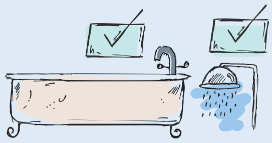

Does it matter whether I have a bath or a shower? No, you can have a bath or a shower – whichever you prefer, as long as it is 6 or more times per week. You don't need to do the same each time.

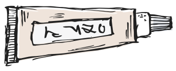

Can I still use my eczema treatments? Yes, you can still use your eczema treatments as often as you need to. If possible, do not start any new eczema treatments during the study.

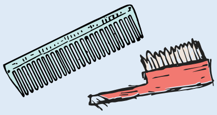

Do I have to wash my hair every time I am in the bath or shower? No, you don't.

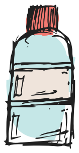

Does it matter what products I use in the bath or shower or after a bath or shower? No. Try to stick to what you would usually use.

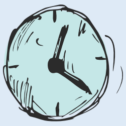

Does it matter how long I take in the bath or shower? No. Try as much as possible to stick to how long you would usually take.

Common Concerns

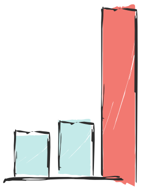

What if my eczema gets worse or flares up during the study? Eczema will naturally change over time. You can keep treating the eczema the way you normally would during a flare up. If you have any concerns, speak to your GP, pharmacist or dermatologist as you usually would.

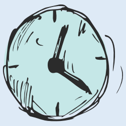

What if I don't have time? You know what works best for you. Your baths or showers don't need to take very long, and you may even find the new routine helpful or enjoyable. Planning ahead may help to fit your baths or showers around your day.

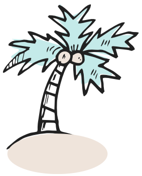

What if I'm staying away from home? Try to stick to your routine as best you can.

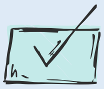

What if I end up having fewer than 6 baths or showers in a week, do I need to leave the study? No, it is very important that you stay in the study and fill in the questionnaires you are sent. This is helpful even if you don't manage to stick to 6 or more baths or showers per week. Just let us know in your answers, so we know if you have been able to stick to having a bath or shower 6 or more times per week.

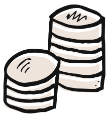

What if I am worried about extra costs from taking part in the study? We have support available to help cover costs if needed.

To access financial support, go to:
https://rc.nctu.ac.uk/surveys/?s=MJJHHPAEFTX894K3

Or scan the QR code:

FUNDED BY NIHR | National Institute for Health and Care Research

This project is funded by the National Institute for Health and Care Research (NIHR) under its Programme Grants for Applied Research programme (PGfAR NIHR203279). The views expressed are those of the author(s) and not necessarily those of the NIHR or the Department of Health and Social Care

Eczema Bathing Study

Thank you for taking part.

You are ready to go!

You are in the Weekly Bathing group…

bath or shower 1 or 2 times per week.

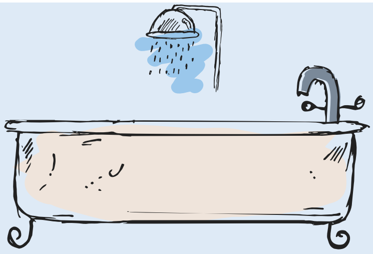

You are in the group that will have a bath or shower 1 or 2 times per week (at most) for the next 4 weeks. A bath or shower includes any way of washing that soaks your body in water.

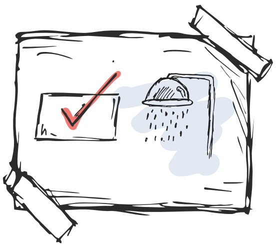

It is important to stick to having a bath or shower no more than 1 or 2 times per week.

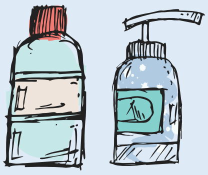

You don’t need to change anything else about the way you wash or manage your eczema.

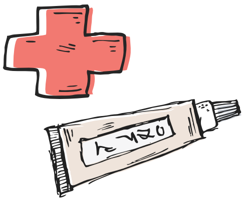

This does not affect your usual eczema care. You can consult your GP, pharmacist or other care provider as usual. If possible, do not start any new eczema treatments for the next 4 weeks.

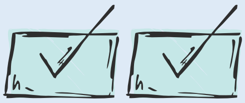

You will fill in questionnaires every week. These are really important to help understand how you are getting on.

Frequently Asked Questions

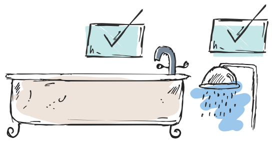

Does it matter whether I have a bath or a shower? No, you can have a bath or a shower – whichever you prefer, as long as it's only 1 or 2 baths/showers per week. You don't need to do the same each time.

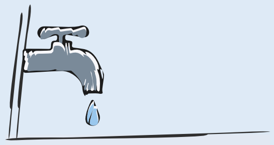

Can I still wash my hands and face every day? Yes, you can wash your hands and face as often as you need to.

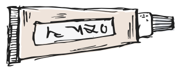

Can I still use my eczema treatments? Yes, you can use your eczema treatments as often as you need to. If possible, do not start any new eczema treatments for the next 4 weeks.

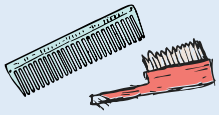

Can I still wash my hair or body without taking a bath or shower? Yes, you can wash your hair or body on 'non-bath/shower' days if you need to. Some people wash their body at a sink for example by using a flannel, or wash their hair by leaning over a bath, sink or shower. The important thing is to not soak your skin in water.

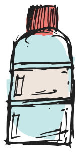

Does it matter what products I use in the bath or shower or after my bath or shower? No. Try as much as possible to stick to what you would usually use.

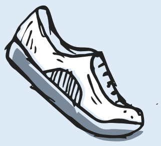

Can I have a shower or bath after playing sport or going swimming? You should only bath/shower 1 or 2 times per week, so it is a good idea to time this for after you play sport or go swimming. If you exercise more often you can try washing in the sink if needed.

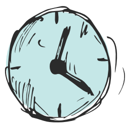

Does it matter how long I take in the bath or shower? No. Try as much as possible to stick to how long you would usually take.

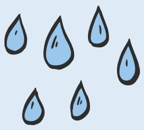

Can I choose to have no baths or no showers at all? Yes, it is fine to wash without baths or showers if you prefer.

Common Concerns

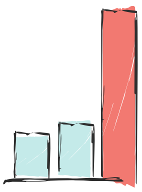

What if my eczema gets worse or flares up during the study? Eczema will naturally change over time. You can keep treating the eczema the way you normally would during a flare up. If you have any concerns, speak to your GP, pharmacist or dermatologist as you usually would.

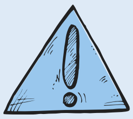

Will people think I am unhygienic, or will I smell? If this is a new routine for you, you might be feeling worried about staying clean. The good news that there are plenty of ways to stay clean without using the bath or shower. Other people are unlikely to notice. Lots of people don't have a bath or shower every day. You can try washing in the sink with a flannel. The important thing is to not soak your skin in water.

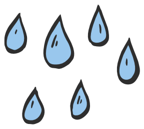

What if I get particularly dirty and feel I need to go in the shower or bath? Try and plan to have baths or showers when you are likely to get dirty. If this is not possible, try washing in the sink. You might find that is enough.

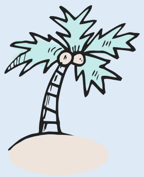

What if I am staying away from home? Try to stick to your routine as best you can.

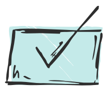

What if I end up having more baths or showers than 1 or 2 times per week? Do I need to leave the study? No, it is very important that you stay in the study and fill in the questionnaires you are sent. This is helpful even if you don't manage to stick to 1 or 2 baths or showers per week. Just let us know in your answers, so we know if you have been able to stick to having a bath or shower 1 or 2 times per week.

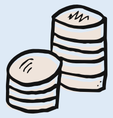

What if I am worried about extra costs from taking part in the study? We have support available to help cover costs if needed.

To access financial support, go to:
https://rc.nctu.ac.uk/surveys/?s=MJJHHPAEFTX894K3

Or scan the QR code:

FUNDED BY NIHR | National Institute for Health and Care Research

This project is funded by the National Institute for Health and Care Research (NIHR) under its Programme Grants for Applied Research programme (PGfAR NIHR203279). The views expressed are those of the author(s) and not necessarily those of the NIHR or the Department of Health and Social Care
