# Supplementary material for: Co-producing a randomized controlled trial on the frequency of bathing in eczema: description of a citizen science approach
Source: Skin Health Dis. 2025 Apr 16;5(2):130–9. doi: 10.1093/skinhd/vzaf005 (PMC12068486; doi:10.1093/skinhd/vzaf005)
Supplement: vzaf005_Supplementary_Data [file vzaf005_supplementary_data.zip › Supplemental Table 1.docx]

**Supplemental Table 1** Structure of prioritisation meetings

| **Meeting** | **Activities** |
| --- | --- |
| **1^st^** | - Introductions - One-to-one breakout discussions - Agreement of ways for working - Format and timing of future meetings - Identification of training needs - Review of initial work and research prioritisation survey (Survey 1) |
| **2^nd^** | - Delivery of RCT training - Explanation of clinical trial concepts and randomisation - Microsoft Teams training - Discussion on research question prioritisation - Review of existing research evidence on the most popular questions from Survey 1 |
| **3^rd^** | - Summary of data from previous eczema studies related to both bathing practices and frequency of bathing - Training on how to design a survey suitable for informing trial design - Development of specific research questions based on Survey 1 results - Preparation for meeting with healthcare professionals |
| **4^th^** | - Presentation of progress to date by nominated person with eczema - Discussion with healthcare professionals about the clinical relevance of the research questions under consideration - Formulation of top two research questions, using the PICO* framework |
| **5^th^** | - Reflection on previous meeting - Review of evidence base for top two priority questions - Decision to proceed with top two voted research questions - Agreement on exact wording of research questions |

*PICO = Population, Intervention, Comparator, Outcomes.
