# Supplementary material for: Co-producing a randomized controlled trial on the frequency of bathing in eczema: description of a citizen science approach
Source: Skin Health Dis. 2025 Apr 16;5(2):130–9. doi: 10.1093/skinhd/vzaf005 (PMC12068486; doi:10.1093/skinhd/vzaf005)
Supplement: vzaf005_Supplementary_Data [file vzaf005_supplementary_data.zip › Supplemental Table 2.docx]

**Supplemental Table 2** Structure of intervention development meetings

| **Meeting** | **Activities** |
| --- | --- |
| **1^st^** | - Brief introduction - Agreement of ways for working - Format and timing of future meetings - Identification of training needs - Introduction to intervention development and key principles - Review of draft survey content and wording - Development of pilot survey with focus on accessibility and inclusion |
| **2^nd^** | - Feedback on pilot survey - Revision resulting in shorter and focused survey - Overview of person-based approach - Tips for intervention development - Consensus on essential information for study information leaflets - Agreement on leaflets structure, format and delivery mode |
| **3^rd^** | - Review of initial versions of study information leaflets - Discussion with healthcare professional on study design aspects relevant to the intervention |
| **4^th^** | - Review of revised study information leaflet - Final refinement of structure and content to enhance clarity, readability and inclusivity |
