# Supplementary material for: Co-producing a randomized controlled trial on the frequency of bathing in eczema: description of a citizen science approach
Source: Skin Health Dis. 2025 Apr 16;5(2):130–9. doi: 10.1093/skinhd/vzaf005 (PMC12068486; doi:10.1093/skinhd/vzaf005)
Supplement: vzaf005_Supplementary_Data [file vzaf005_supplementary_data.zip › Supplemental Table 3.docx]

**Supplemental Table 3** Structure of trial design meetings

| **Meeting** | **Activities** |
| --- | --- |
| **1^st^** | - Training on common terminology used in clinical trials (eligibility criteria, baseline characteristics, stratification, adherence, prognostic factors) - Discussion on inclusion and exclusion criteria for the study |
| **2^nd^** | - Training on the principles of consent and assent in clinical trials - Completion of baseline characteristics questions - Discussion on eligibility screening questions wording - Establishing procedures for obtaining assent and consent across different age groups - Agreement on payment approach for participants in the study to help cover some costs related to bathing more frequently |
| **3^rd^** | - Training on trial outcomes and core outcome sets - Decision on primary outcome measure - Discussion on secondary outcome measures |
| **4^th^** | - Summary of trial design decisions made - Sign-off on study materials created by the intervention development group - Discussion on outstanding issues (study title, frequency of data collection, process evaluation methods) - Further discussion on recruitment methods and timelines, considering ways of raising awareness about the trial |
