# Supplementary material for: Co-producing a randomized controlled trial on the frequency of bathing in eczema: description of a citizen science approach
Source: Skin Health Dis. 2025 Apr 16;5(2):130–9. doi: 10.1093/skinhd/vzaf005 (PMC12068486; doi:10.1093/skinhd/vzaf005)
Supplement: vzaf005_Supplementary_Data [file vzaf005_supplementary_data.zip › Supplemental Table 4.docx]

**Supplemental Table 4** Summary of baseline characteristics of survey respondents

| **Characteristics** | **Prioritisation Survey**  ***n =* 120** | **Intervention Development Survey**  ***n =* 169** |
| --- | --- | --- |
| Age (years), *n* (%) |  |  |
| ≤16 | 19 (16) | 62 (36) |
| 17-25 | 23 (19) | 22 (13) |
| ≥26 | 78 (65) | 83 (49) |
| Prefer not to say | 0 | 1 (1) |
| No response | n/a | 1 (1) |
| Gender, *n* (%) |  |  |
| Female | 35 (29) | 111 (65) |
| Male | 12 (10) | 54 (32) |
| Prefer not to say | 1 (1) | 3 (2) |
| No response | 72 (60) | 1 (1) |
| Ethnicity, *n* (%) |  |  |
| White British | 69 (58) | 120 (71) |
| White Other | 13 (11) | 16 (9) |
| Black, Black British, Caribbean or African | 11 (9) | 7 (4) |
| Asian or Asian British | 15 (12) | 12 (7) |
| Mixed or multiple ethnic groups | 5 (4) | 10 (6) |
| Prefer not to say | 1 (1) | 1 (1) |
| Other | 6 (5) | 2 (1) |
| No response | 0 | 1 (1) |
| Experience of eczema, *n* (%)* |  |  |
| Self-reported eczema | 82 (51) | 107 (63) |
| Carer of someone with eczema | 46 (29) | 62 (37) |
| Healthcare professional | 16 (10) | n/a |
| Researcher | 16 (10) | n/a |

***** Some respondents belonged to multiple categories.
